# Association of behavioral characteristics and mental health indices in a large nationwide representative sample of schoolchildren aged 11-15 years in Albania

**DOI:** 10.3325/cmj.2025.66.125

**Published:** 2025-04

**Authors:** Iris Mone, Alketa Qosja, Gentiana Qirjako, Rudina Çumashi, Enver Roshi, Genc Burazeri

**Affiliations:** 1 Faculty of Medicine, University of Medicine, Tirana, Albania; 2 Institute of Public Health, Tirana, Albania; 3 Department of International Health, CAPHRI (Care and Public Health Research Institute), Maastricht University, Maastricht, the Netherlands

## Abstract

**Aim:**

To assess the independent associations of behavioral factors with many mental health indices among children in Albania.

**Methods:**

This cross-sectional study carried out in Albania in 2022 included a nationwide representative sample of 5454 schoolchildren aged 11, 13, and 15 years (52% girls; response rate: 96%). Data on ten mental health indices were collected, in addition to behavioral characteristics and sociodemographic factors. A general linear model was used to assess the independent associations of behavioral factors with mental health indices.

**Results:**

Irrespective of sociodemographic characteristics, the scores of all mental health indices were significantly worse among schoolchildren who reported lifetime smoking and alcohol consumption, those who engaged less frequently in physical activity, those who skipped breakfast at least on one weekday, and those who consumed fresh fruit less than daily compared with their counterparts who were, respectively, never smokers, had never drunk alcohol, were more physically active, consumed breakfast regularly, and consumed fresh fruit daily.

**Conclusions:**

Irrespective of sociodemographic boundaries, this study showed significant associations between behavioral characteristics and the mental health of Albanian schoolchildren. This highlights the importance of promoting healthy behaviors among children to mitigate the risk of mental health challenges. Interventions aimed at encouraging regular physical activity, consistent breakfast consumption, and daily intake of fresh fruit may enhance the mental resilience of school-aged children.

In 2019, about 8% of children aged 5-9 years and 14% of those aged 10-19 years globally suffered from a mental disorder (1-3). The most common mental disorder in children aged <5 years is developmental disability, followed by autism spectrum disorder (which constitutes another developmental disorder) (1). The frequency of these conditions decreases over time, as a significant proportion of individuals with developmental disorders die at a young age (1). Regarding the age-group 10-14 years, attention-deficit (and/or hyperactivity) disorder and conduct disorders are especially common among younger boys (1). Conversely, the most prevalent mental disorder among older adolescents is anxiety (4.6%), particularly among adolescent girls (5.5%) (1-3). Notably, half of the mental disorders present in adulthood develop by the age of 14 years, and three quarters by the age of 24 years (4).

Besides a range of structural, family, and community factors, individual factors are also important determinants of mental health, including genetic factors, social and emotional skills, sense of mastery, physical health, sleep disturbances, diet, overweight and obesity, substance use, and physical activity (1,5,6). These factors apply among both children and adolescents, increasing their vulnerability for developing mental health disorders (1,5,6). Mental health problems make children much more vulnerable to becoming victims of violence (7). Furthermore, anxiety and depressive disorders among children may be associated with bullying victimization (1), along with eating disorders, which are more prevalent among girls (8).

Health Behavior in School-aged Children (HBSC) is a wide-ranging school-based survey, which, since mid-1980s, has been conducted every four years in many European countries and beyond in collaboration with the World Health Organization (WHO) Regional Office for Europe (9,10). The HBSC surveys collect information about an array of health behavioral practices, health outcomes, and social environments of children aged 11, 13, and 15 years (9,10).

Albania is a Southeastern European country that emerged in 1990 from the most rigid communist regime (11,12). Since then, Albania has been undergoing profound changes and deep political and socioeconomic reforms, which, nevertheless, have been characterized by periodic civil unrest and social upheaval (11,12).

The first round of the HBSC survey in Albania was conducted in 2009-2010 (13), whereas the last (fourth) round was carried out in 2022, in parallel with 43 other countries in Europe, Central Asia, and Canada (9,10). In addition to other health outcomes and behavioral practices, the survey encompassed a series of questions aimed at measuring self-reported mental health status of schoolchildren aged 11, 13, and 15 years (14).

While mental health issues among adolescents are increasingly recognized globally (1), data from the Western Balkans, including Albania, remain particularly sparse, fragmented, and often outdated. Existing literature in this context is often limited to small-scale studies that lack representative data and culturally relevant instruments. Additionally, regional socio-political transitions, economic instability, and evolving social norms may uniquely shape behavioral patterns and psychosocial stressors among adolescents in Albania and other countries of the Southeastern European region.

In this context, the aim of our study was to assess the independent associations of a wide array of mental health indices with behavioral characteristics of Albanian schoolchildren included in the last HBSC round conducted in 2022. We hypothesized worse scores of mental health indices among schoolchildren with unhealthy behavioral practices, regardless of their sociodemographic characteristics.

## Participants and methods

### Study design

HBSC is a cross-sectional study based on an internationally standardized instrument (14), validated in Albania since 2009-2010 (13).

### Study population and sampling

The study sample was drawn based on the HBSC international protocol (14), consisting of a nationwide stratified multistage cluster sample with probability proportional to size. According to the HBSC protocol, the minimum required sample size was 4650 pupils (1550 children for each age-group: 11, 13, and 15 years) (14). We drew a larger sample consisting of 5700 pupils to increase the study power, considering also potential non-response.

Of the 5700 targeted schoolchildren, 246 refused to participate and/or provided incomplete or non-valid information. Ultimately, the study sample included 5454 schoolchildren aged 11, 13, and 15 years (2844 girls, or 52% of the whole sample), with an overall response rate of 96%. Written informed consent from the participants’ legal guardian/next of kin was not required for study participation in accordance with the national legislation and the institutional requirements. The ethics committee has waived the requirement for written informed consent to participate from the participants’ legal guardians in line with the guidelines and recommendations envisaged in the HBSC protocol.

### Instrument

A structured self-administered questionnaire gathered information on mental health indices, in addition to behavioral characteristics and sociodemographic factors of schoolchildren. The data collection instrument is described in detail in the international HBSC protocol (14).

In short, we measured the following ten indices (9,14): self-rated health, selected health complaints (feeling low, irritability, nervousness, and sleep disorders), loneliness, WHO-5 well-being index (in this study referred to as “mood”), self-efficacy, Generalized Anxiety Disorder (GAD-7) (in this study referred to as “anxiety”), and COVID-19 impact on mental health. Behavioral characteristics consisted of lifetime smoking and alcohol consumption, physical activity, breakfast consumption on weekdays, and consumption of fresh fruit (14). Sociodemographic factors included sex, place of residence, mother’s and father’s current employment status, and family affluence scale (14).

The international HBSC instrument was validated in Albania before the first survey round was carried out in 2009-2010 (13). After translation (from English into Albanian) and back-translation (from Albanian into English) of the survey questionnaire, the validation process consisted of the assessment of psychometric properties of different modules of the instrument (behavioral characteristics and mental health), including assessment of internal consistency and test-retest reliability (stability over time).

### Statistical analysis

A Fisher exact test was employed to assess the differences in the distribution of behavioral factors (lifetime smoking and alcohol intake, physical activity, breakfast consumption, and fruit consumption) among schoolchildren aged 11, 13, and 15 years (Table 1). To facilitate analytical simplicity and communication of findings to broader public health audiences, all behavioral factors were also dichotomized in line with the international HBSC protocol (14).

**Table 1:** Age-specific distribution of behavioral factors in a nationwide representative sample of Albanian schoolchildren aged 11-15 years included in the HBSC 2022 survey (N = 5446)*

Behavioral factors	11 years (N = 1784)	13 years (N = 1785)	15 years (N = 1877)	P^†^
**Lifetime smoking:**				<0.001
Never	1677 (95.7)	1560 (89.7)	1438 (78.8)	
≥1-2 days/lifetime	76 (4.3)	180 (10.3)	387 (21.2)	
Total	1753 (100.0)	1740 (100.0)	1825 (100.0)	
**Lifetime alcohol consumption:**				<0.001
Never	1587 (91.2)	1387 (80.5)	1084 (60.1)	
≥1-2 days/lifetime	154 (8.8)	335 (19.5)	719 (39.9)	
**Physical activity:**				<0.001
≤2 times/week	494 (28.1)	613 (34.9)	856 (45.9)	
≥3 times/week	1262 (71.9)	1142 (65.1)	1007 (54.1)	
**Breakfast:**				<0.001
≤4 weekdays	623 (37.8)	898 (54.4)	1118 (62.1)	
5 weekdays	1023 (62.2)	754 (45.6)	681 (37.9)	
**Fruit consumption:**				<0.001
Less than daily	624 (35.3)	757 (43.0)	842 (45.2)	
Daily	1145 (64.7)	1004 (57.0)	1021 (54.8)	

*Absolute numbers and their respective column percentages (in parentheses). There were eight missing values for the age of schoolchildren who participated in the HBSC survey. Discrepancies in the totals are due to the following missing values for behavioral factors: smoking (n = 128), alcohol consumption (n = 180), physical activity (n = 72), breakfast consumption (n = 349), and fruit consumption (n = 53).

**†**Fisher exact test.

Conversely, a general linear model was used to compare the mean values of mental health indices (expressed as numerical terms) by behavioral characteristics of schoolchildren. The general linear model procedure provides regression analysis and analysis of variance for one dependent variable by one or more factors (variables). Initially, crude (unadjusted) mean values and their respective *P* values were calculated (Table 2). Subsequently, general linear models were adjusted for sociodemographic characteristics of schoolchildren (age, sex, place of residence, parental employment status, and family affluence scale). Multivariable-adjusted mean values and their respective *P* values were calculated (Table 3).

**Table 2:** Crude (unadjusted) mean values of mental health indices by behavioral characteristics of Albanian schoolchildren aged 11-15 years, HBSC 2022 survey

Behavioral factors	General health status and health complaints *									
	Self-rated health		Feeling low		Irritability		Nervousness		Sleep difficulties	
	Mean ^†^	P	Mean	P	Mean	P	Mean	P	Mean	P
**Total sample**	3.67	-	3.51	-	3.54	-	3.60	-	4.18	-
**Smoking**		<0.001		<0.001		<0.001		<0.001		<0.001
Never	3.70		3.59		3.65		3.70		4.28	
≥1-2 days/lifetime	3.47		3.00		2.86		2.93		3.59	
**Alcohol consumption**		<0.001		<0.001		<0.001		<0.001		<0.001
Never	3.71		3.64		3.73		3.77		4.31	
≥1-2 days/lifetime	3.56		3.07		2.94		3.04		3.80	
**Physical activity**		<0.001		<0.001		<0.001		<0.001		<0.001
≤2 times/week	3.55		3.24		3.27		3.35		4.00	
≥3 times/week	3.74		3.67		3.70		3.74		4.28	
**Breakfast**		<0.001		<0.001		<0.001		<0.001		<0.001
≤4 weekdays	3.60		3.27		3.29		3.34		4.02	
5 weekdays	3.75		3.75		3.80		3.87		4.37	
**Fruit consumption**		<0.001		<0.001		<0.001		<0.001		<0.001
Less than daily	3.59		3.31		3.36		3.41		4.03	
Daily	3.73		3.64		3.67		3.73		4.29	
**Behavioral factors**	Other mental health indices*									
	**Loneliness**		**WHO-5 index (“mood”)**		**Self-efficacy**		**Anxiety** **(GAD-7)**		**COVID-19 impact**	
	**Mean^†^**	**P**	**Mean**	**P**	**Mean**	**P**	**Mean**	**P**	**Mean**	**P**
**Total sample**	4.00	-	17.9	-	7.89	-	15.7	-	3.02	-
**Smoking**		<0.001		<0.001		0.076		<0.001		<0.001
Never	4.06		18.2		7.91		16.1		3.05	
≥1-2 days/lifetime	3.61		15.8		7.78		13.3		2.82	
**Alcohol consumption**		<0.001		<0.001		0.738		<0.001		<0.001
Never	4.10		18.4		7.91		16.3		3.07	
≥1-2 days/lifetime	3.69		16.2		7.89		13.8		2.87	
**Physical activity**		<0.001		<0.001		<0.001		<0.001		<0.001
≤2 times/week	3.79		16.2		7.59		14.5		2.89	
≥3 times/week	4.13		18.9		8.08		16.4		3.09	
**Breakfast**		<0.001		<0.001		<0.001		<0.001		<0.001
≤4 weekdays	3.80		16.9		7.65		14.6		2.93	
5 weekdays	4.20		19.0		8.18		16.8		3.13	
**Fruit consumption**		<0.001		<0.001		<0.001		<0.001		<0.001
Less than daily	3.86		16.8		7.51		15.0		2.89	
Daily	4.10		18.7		8.16		16.2		3.11	

*Missing values for mental health indices: self-rated health (n = 22), feeling low (n = 264), irritability (n = 306), nervousness (n = 314), sleep difficulties (n = 325), loneliness (n = 21), WHO-5 index (n = 332), self-efficacy (n = 147), anxiety (n = 290), and COVID-19 impact on mental health (n = 195).

**†**Mean values and *P* values from the general linear model. Lower mean values indicate worse indices for all ten mental health variables (five of which are presented in the upper panel, whereas the other five are presented in the lower panel of the table).

**Table 3:** Multivariable-adjusted mean values of mental health indices by behavioral characteristics of Albanian schoolchildren aged 11-15 years, HBSC 2022 survey

Behavioral factors	General health status and health complaints											
	Self-rated health		Feeling low		Irritability		Nervousness			Sleep difficulties		
	Mean*	P	Mean	P	Mean	P	Mean		P	Mean		P
**Smoking**		<0.001		<0.001		<0.001			<0.001			<0.001
Never	3.64		3.50		3.59		3.64			4.23		
≥1-2 days/lifetime	3.42		2.99		2.94		2.99			3.61		
**Alcohol consumption**		<0.001		<0.001		<0.001			<0.001			<0.001
Never	3.65		3.55		3.67		3.71			4.26		
≥1-2 days/lifetime	3.51		3.06		3.01		3.11			3.81		
**Physical activity**		<0.001		<0.001		<0.001			<0.001			<0.001
≤2 times/week	3.51		3.27		3.35		3.41			4.02		
≥3 times/week	3.67		3.54		3.60		3.64			4.23		
**Breakfast**		<0.001		<0.001		<0.001			<0.001			<0.001
≤4 weekdays	3.56		3.27		3.36		3.38			4.03		
5 weekdays	3.68		3.63		3.71		3.77			4.31		
**Fruit consumption**		<0.001		<0.001		<0.001			<0.001			<0.001
Less than daily	3.54		3.26		3.35		3.39			4.01		
Daily	3.68		3.57		3.63		3.68			4.26		
**Behavioral factors**	**Other mental health indices**											
	**Loneliness**		**WHO-5 index (“mood”)**		**Self-efficacy**			**Anxiety** **(GAD-7)**		**COVID-19 impact**		
	Mean *	**P**	**Mean**	**P**	**Mean**	**P**		**Mean**	**P**	**Mean**	**P**	
**Smoking**		<0.001		<0.001		0.033			<0.001		<0.001	
Never	4.00		18.2		7.85			15.9		3.00		
≥1-2 days/lifetime	3.62		16.2		7.68			13.5		2.79		
**Alcohol consumption**		<0.001		<0.001		0.137			<0.001		<0.001	
Never	4.03		18.3		7.86			16.1		3.02		
≥1-2 days/lifetime	3.70		16.7		7.76			14.0		2.85		
**Physical activity**		<0.001		<0.001		<0.001			<0.001		<0.001	
≤2 times/week	3.84		16.7		7.50			14.9		2.86		
≥3 times/week	4.03		18.6		8.00			16.0		3.04		
**Breakfast**		<0.001		<0.001		<0.001			<0.001		<0.001	
≤4 weekdays	3.82		17.3		7.57			14.8		2.91		
5 weekdays	4.12		18.7		8.09			16.5		3.07		
**Fruit consumption**		<0.001		<0.001		<0.001			<0.001		<0.001	
Less than daily	3.82		16.9		7.46			14.9		2.86		
Daily	4.06		18.7		8.08			16.1		3.07		

*Mean values and *P* values from the general linear model adjusted for the following sociodemographic characteristics: age, sex, place of residence, father’s employment status, mother’s employment status, and family affluence scale. Lower mean values indicate worse indices for all ten mental health variables (five of which are presented in the upper panel, whereas the other five are presented in the lower panel of the table).

Furthermore, correlation coefficients of mental health indices and behavioral characteristics of schoolchildren were calculated (Table 4). Initially, crude/unadjusted Spearman’s correlation coefficients and their respective *P* values were calculated. Next, partial correlation coefficients were calculated adjusting simultaneously for all sociodemographic factors (age, sex, place of residence, parental employment status, and family affluence scale). Partial correlation coefficients measure the degree of linear association between variables while controlling for the effects of one or more additional variables (potential confounders). Based on this feature, multivariable-adjusted correlation coefficients and their respective *P* values were calculated. For all statistical tests used, *P* ≤ 0.05 was considered as statistically significant. SPSS (IBM Corp., Armonk, NY, USA), version 19.0, was used for all the statistical analyses.

**Table 4:** Correlation coefficients of mental health indices and behavioral characteristics

Crude (unadjusted) correlation coefficients					
Mental health indices	Behavioral factors				
	Smoking	Alcohol consumption	Physical activity	Breakfast consumption	Fruit consumption
**Self-rated health**	-0.12 (<0.001)*	-0.11 (<0.001)	0.18 (<0.001)	0.14 (<0.001)	0.13 (<0.001)
**Feeling low**	-0.13 (<0.001)	-0.17 (<0.001)	0.16 (<0.001)	0.16 (<0.001)	0.13 (<0.001)
**Irritability**	-0.17 (<0.001)	-0.23 (<0.001)	0.15 (<0.001)	0.18 (<0.001)	0.12 (<0.001)
**Nervousness**	-0.16 (<0.001)	-0.22 (<0.001)	0.15 (<0.001)	0.19 (<0.001)	0.13 (<0.001)
**Sleep difficulties**	-0.17 (<0.001)	-0.18 (<0.001)	0.13 (<0.001)	0.14 (<0.001)	0.11 (<0.001)
**Loneliness**	-0.12 (<0.001)	-0.15 (<0.001)	0.17 (<0.001)	0.19 (<0.001)	0.11 (<0.001)
**WHO-5 index**	-0.13 (<0.001)	-0.18 (<0.001)	0.27 (<0.001)	0.21 (<0.001)	0.19 (<0.001)
**Self-efficacy**	-0.02 (0.154)	-0.01 (0.712)	0.17 (<0.001)	0.14 (<0.001)	0.23 (<0.001)
**Anxiety (GAD-7)**	-0.17 (<0.001)	-0.22 (<0.001)	0.19 (<0.001)	0.24 (<0.001)	0.15 (<0.001)
**COVID-19 impact**	-0.06 (<0.001)	-0.06 (<0.001)	0.06 (<0.001)	0.09 (<0.001)	0.09 (<0.001)
**Partial correlation coefficients**					
**Mental health indices**	**Behavioral factors**				
	**Smoking**	**Alcohol consumption**	**Physical activity**	**Breakfast consumption**	**Fruit consumption**
**Self-rated health**	-0.12 (<0.001) **^†^**	-0.11 (<0.001)	0.16 (<0.001)	0.12 (<0.001)	0.13 (<0.001)
**Feeling low**	-0.11 (<0.001)	-0.14 (<0.001)	0.13 (<0.001)	0.13 (<0.001)	0.11 (<0.001)
**Irritability**	-0.13 (<0.001)	-0.19 (<0.001)	0.10 (<0.001)	0.13 (<0.001)	0.11 (<0.001)
**Nervousness**	-0.13 (<0.001)	-0.17 (<0.001)	0.10 (<0.001)	0.12 (<0.001)	0.11 (<0.001)
**Sleep difficulties**	-0.12 (<0.001)	-0.15 (<0.001)	0.10 (<0.001)	0.11 (<0.001)	0.10 (<0.001)
**Loneliness**	-0.10 (<0.001)	-0.12 (<0.001)	0.11 (<0.001)	0.14 (<0.001)	0.11 (<0.001)
**WHO-5 index**	-0.11 (<0.001)	-0.13 (<0.001)	0.23 (<0.001)	0.15 (<0.001)	0.19 (<0.001)
**Self-efficacy**	-0.01 (0.526)	-0.01 (0.439)	0.17 (<0.001)	0.13 (<0.001)	0.21 (<0.001)
**Anxiety (GAD-7)**	-0.16 (<0.001)	-0.18 (<0.001)	0.15 (<0.001)	0.18 (<0.001)	0.15 (<0.001)
**COVID-19 impact**	-0.03 (0.042)	-0.04 (0.012)	0.07 (<0.001)	0.08 (<0.001)	0.09 (<0.001)

*Spearman’s correlation coefficients and their respective *P* values (in parentheses). Negative correlations between mental health indices with smoking and alcohol consumption indicate inverse linear associations between these two unhealthy habits with good mental health. Conversely, positive correlations between mental health indices with physical activity, breakfast consumption and fruit consumption indicate positive linear associations between these three healthy behaviors with good mental health.

**†**Correlation coefficients adjusted for sociodemographic characteristics of schoolchildren (age, sex, place of residence, father’s employment status, mother’s employment status, and family affluence scale).

## Results

Overall, 21% of 15-year-old children reported lifetime smoking compared with 10% of 13-year-old and only 4% of 11-year-old children (*P* < 0.001) (Table 1). Furthermore, 40% of 15-year-old children reported lifetime alcohol consumption compared with 20% of 13-year-old and 9% of 11-year-old children (*P* < 0.001). Conversely, 11-year-old children reported more frequent (≥3 times/week) physical activity (72%) compared with 13-year-olds (65%) and older children (54%) (*P* < 0.001). A similar age-trend was evident for breakfast consumption, with 62% of the youngest children reporting breakfast consumption on all five weekdays compared with 46% of 13-year-olds and 38% of 15-year-olds (*P* < 0.001). Also, daily consumption of fresh fruit was highest among the youngest children (65%) compared with 13- and 15-year-olds (57% and 55%, respectively; *P* < 0.001) (Table 1).

**Figure 1:**
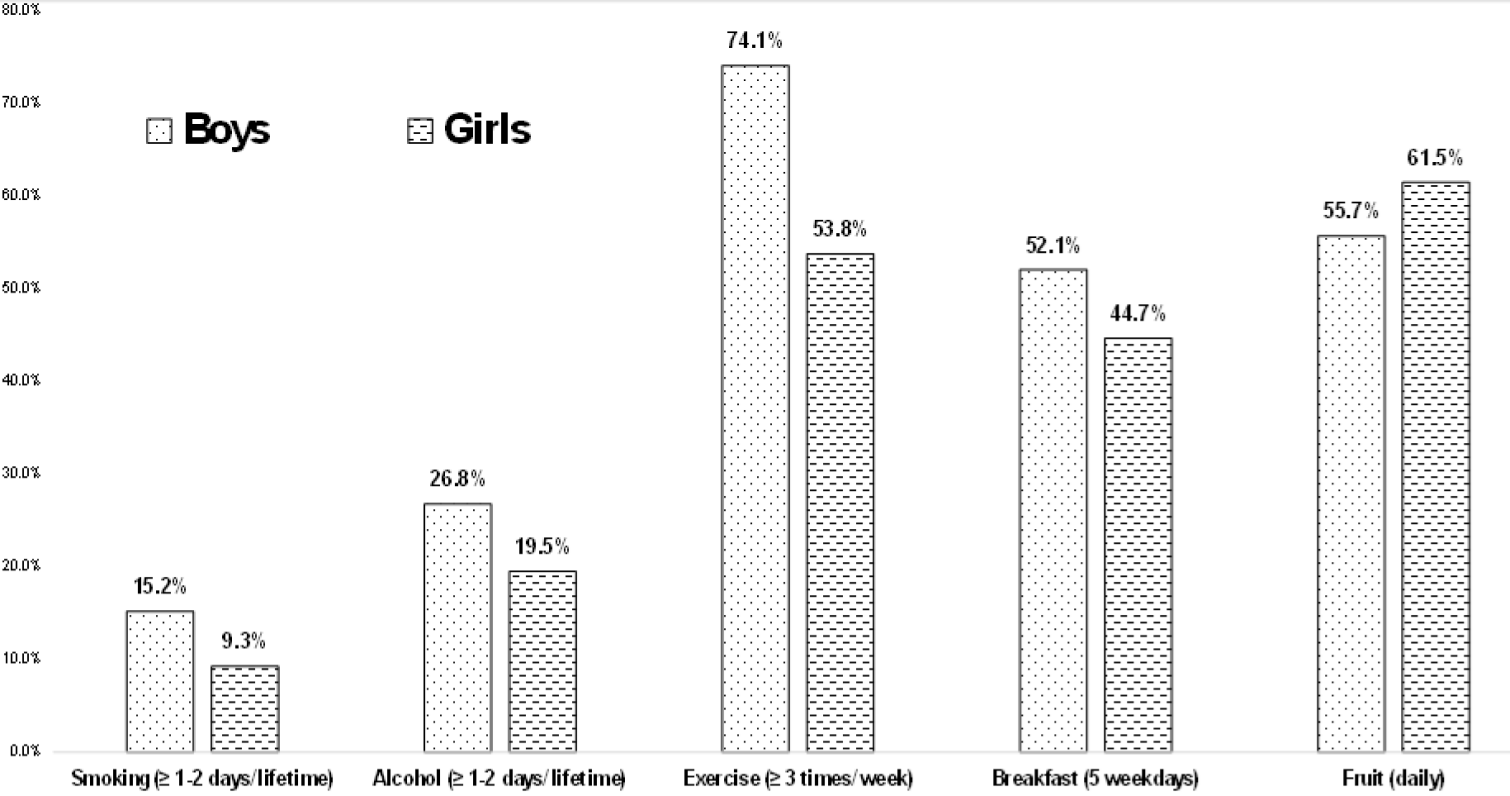
Distribution of behavioral characteristics by schoolchildren’s sex (N = 5446).

Regarding sex differences (Figure 1), the prevalence of smoking and alcohol consumption was higher in boys (15% vs 9% and 27% vs 20%). Additionally, breakfast consumption and adequate physical exercise were more frequent in boys (52% vs 45% and 74% vs 54%), whereas the opposite was evident for fruit intake (56% vs 62%).

Crude (unadjusted) mean values of mental health indices were all lower (indicating a poorer mental health status) among children who reported lifetime smoking compared with never smokers (Table 2). Similarly, except for self-efficacy, the mean values of mental health indices were all lower among children who reported lifetime alcohol consumption compared with never drinkers. Likewise, the mean values of mental health indices were all lower among less physically active children, those who skipped breakfast at least for one weekday, and children who consumed fresh fruit less than daily compared with their counterparts who were, respectively, more physically active, consumed breakfast regularly, and ate fresh fruit daily (Table 2). Remarkably, across all behavioral factors, the differences in mean scores were not only significant (*P* < 0.001) but also practically meaningful, suggesting clear patterns with potential implications for public health interventions. Thus, negative health behaviors (smoking and alcohol use) were consistently associated with poorer mental health outcomes: schoolchildren who had tried smoking or alcohol even minimally reported worse self-rated health and higher frequencies of emotional and somatic complaints. For example, those who had smoked at least once had a lower mean self-rated health score (3.5 vs 3.7) and markedly lower mental health indicators, including a mean score for feeling low at 3.0 compared with 3.6 in non-smokers – a difference large enough to suggest substantial impact at the population level. Conversely, healthy behaviors (frequent physical activity, regular breakfast, and daily fruit intake) were associated with notably better perceived health and fewer psychological complaints. For instance, schoolchildren active ≥3 times/week reported higher self-rated health (3.7 vs 3.5) and significantly fewer emotional symptoms. These consistent gradients, even in modestly spaced categories (like “≤2 times/week” vs “≥3 times/week”), underscore the potential for even small lifestyle changes to yield tangible improvements in adolescent mental health.

Even after adjustment for all sociodemographic characteristics (Table 3), the mean values of mental health indices were all lower among children who reported lifetime smoking and lifetime alcohol consumption (except for self-efficacy), among less physically active children, those who skipped breakfast at least for one weekday, and children who consumed fresh fruit less than daily compared with their counterparts who were, respectively, never smokers, had never drunk alcohol, were more physically active, consumed breakfast regularly, and consumed fresh fruit daily (Table 3).

There were weak but significant bivariate correlations (Table 4) of most mental health indices with behavioral characteristics. Adjustment for sociodemographic factors slightly attenuated the correlation coefficients, especially those pertinent to the linear associations of mental health indices with physical activity and breakfast consumption. The range of linear associations varied from almost non-existent correlations for the COVID-19 impact on mental health to weak-to-moderate correlations between the following measures: physical activity and WHO-5 well-being index (partial r = 0.23); fruit consumption and self-efficacy (partial r = 0.21), or WHO-5 well-being index (partial r = 0.19); alcohol consumption and irritability (partial r = -0.19), or anxiety (partial r = -0.18); and breakfast consumption with anxiety (partial r = 0.18) (Table 4).

## Discussion

The main findings of our study include consistent associations of mental health indices with several behavioral factors of schoolchildren, irrespective of a range of sociodemographic characteristics including sex, place of residence, parental employment status, and family affluence. Hence, upon multivariable-adjustment for key sociodemographic characteristics, the scores of all mental health indices were significantly worse among schoolchildren who reported lifetime smoking and/or alcohol consumption, those who were engaged less frequently in physical activity, those who skipped breakfast at least for one weekday, and those who consumed fresh fruit less than daily compared with their counterparts who were, respectively, never smokers, had never drunk alcohol, were more physically active, consumed breakfast regularly, and consumed fresh fruit daily.

Our findings on the links between unhealthy behaviors and poorer mental health are compatible with many reports from the international literature (1,5,6,15,16). Thus, several studies (15,16) have indicated that an array of mental health problems (including depression, anxiety, and stress-related disorders) is related to unhealthy behaviors, such as unhealthy dietary habits, poor sleep quality, physical inactivity, and higher levels of tobacco smoking (15,16). Notably, results from population-based studies indicate that the links between behavioral risk factors and mental conditions endure in low- and middle-income countries too (15,17-19).

More specifically, our findings on physical activity are in line with the international literature (15-17), as we documented a higher score on anxiety levels and a higher score on sleep difficulties among less physically active schoolchildren.

Furthermore, compatible with several international reports (6,18), our findings indicate that the scores of all mental health indices were significantly worse among those who reported lifetime smoking and/or alcohol consumption.

The positive association of good mental health with healthy nutritional habits (breakfast consumption on all days, and a higher fruit consumption) evidenced in our study is compatible with previous reports (1,6). However, poorer nutritional habits among children may serve as a marker of a lower socioeconomic status, which, in turn, has been convincingly linked to poorer mental health (1,6). Indeed, children and adolescents from lower socioeconomic backgrounds experience greater exposure to poor environments and stressful family contexts, which leads to poorer mental health (6,20). In addition, anxiety and depressive disorders among children are related to eating disorders (1,21). Yet, in our study, the positive association between better mental health and healthy dietary habits persisted upon adjustment for a range of sociodemographic factors.

In our study, the varying strength of associations observed between behavioral factors and mental health outcomes may reflect underlying cultural, social, and educational dynamics specific to the Albanian context. Regular breakfast consumption or physical activity showed stronger positive associations with mental health probably because they are embedded in daily routines that are influenced by family structure, school engagement, and parental monitoring – factors that remain relatively strong in Albanian society. In contrast, smoking and alcohol use, although less normative in early adolescence, may reflect more complex psychosocial stressors such as peer pressure, or early exposure to risk behaviors in the Albanian environment with limited mental health literacy and prevention infrastructure. Furthermore, the stigma around mental health and limited access to school-based psychological support may lead Albanian schoolchildren to internalize distress, which makes associations with substance use or poor nutrition particularly pronounced as coping mechanisms. These contextual elements pertinent to the Albanian environment may amplify or buffer the impact of certain behaviors on mental health, which underscores the need for culturally grounded interpretations and interventions.

In the Albanian and the Western Balkans context – where preventive youth mental health services are underdeveloped – our findings suggest that school-based and community-based health promotion programs targeting diet, physical activity, and substance use could measurably affect both emotional well-being and general health perceptions of children. Therefore, there is a strong case for investing in behavioral interventions as an efficient and scalable mental health strategy.

The strengths of this study include a large, nationally representative sample with a high response rate; application of a standardized and validated international survey tool; inclusion of a broad array of mental health indicators; and a comprehensive adjustment for key sociodemographic confounders.

Study limitations encompass several factors. First, our findings might not be applicable to Albanian children of the same age who are not enrolled in formal education, but may differ in socioeconomic status, health behaviors, or mental health indices compared with their in-school peers, which possibly leads to underrepresentation of more marginalized or at-risk subgroups. Second, there are potential information biases, which could manifest as underreporting, misunderstanding, or misinterpreting sensitive data, especially concerning measures related to mental health. In particular, over-reporting of unhealthy behaviors among children with poorer mental health could lead to differential misclassification, possibly exaggerating the observed associations. Also, the COVID-19 mental health impact measure served as a proxy for pandemic-related psychological distress, enabling the identification of behavioral correlates that may both reflect and exacerbate children’s vulnerability to mental health challenges during a period of societal isolation. Yet, another limitation concerns the potential for recall or reporting bias due to heightened public and media attention on mental health during the pandemic, which may have influenced how children perceived and reported their psychological experiences. Third, causal relationships cannot be inferred from cross-sectional studies due to the simultaneous measurement of exposures and outcomes. Hence, in the context of our study, the cross-sectional design restricts the capacity to establish temporal precedence – whether certain behavioral factors lead to mental health outcomes, or vice versa. This limitation raises the potential for reverse causality and confounding. Therefore, our findings should be cautiously interpreted, an issue pointing to the need for future research to address these potential constraints and provide a more comprehensive understanding of the links between mental health and behavioral characteristics of children and adolescents.

Our findings bear important policy implications for health professionals and decision-makers in Albania and elsewhere, given the high burden of mental disorders at a global scale, with an estimated number of over 125 million children and adolescents suffering from a mental health problem in 2019 (corresponding to about 13% of the overall population) (1-3,22). Hence, the WHO Regional Office for Europe has envisioned several key strategic objectives and actions in its “European Framework for Action on Mental Health 2021-2025” (22). This framework for action is intended to consolidate existing and emerging evidence to provide support for planning, implementing and tracking mental health policies, programs, and services in the European region (22).

In conclusion, regardless of background, schoolchildren who reported unhealthy behaviors exhibited significantly poorer mental health scores compared with their counterparts with healthier habits. This highlights the critical importance of promoting and supporting healthy behaviors among children to mitigate the risk of mental health challenges. Interventions aimed at discouraging substance use and encouraging regular physical activity, consistent breakfast consumption, and daily intake of fresh fruits hold promise in enhancing the mental resilience of school-aged children, irrespective of their sociodemographic profiles.

## References

[1] World Health Organization. World mental health report: transforming mental health for all. Geneva: World Health Organization; 2022. Available from: https://apps.who.int/iris/handle/10665/356119. Accessed: May 6, 2024.

[2] Institute for Health Metrics and Evaluation. GBD Results Tool; 2019. In: Global Health Data Exchange. Seattle: Institute for Health Metrics and Evaluation. Available from: http://ghdx.healthdata.org/gbd-results-tool?params=gbd-api-2019-permalink/cb9c37d9454c80df77adaed394d7fc0f. Accessed: May 6, 2024.

[3] Institute for Health Metrics and Evaluation. GBD Results Tool; 2019. In: Global Health Data Exchange. Seattle: Institute for Health Metrics and Evaluation; 2019. Available from: http://ghdx.healthdata.org/gbd-results-tool?params=gbd-api-2019-permalink/5066348dc958b095cb6ceb4bfd9c3e07. Accessed: May 6, 2024.

[4] Kessler R Berglund P Demler O Jin R Merikangas K Walters EE Lifetime prevalence and age-of-onset distributions of DSM-IV disorders in the National Comorbidity Survey replication. Arch Gen Psychiatry 2005 62 593 602 10.1001/archpsyc.62.6.593 15939837

[5] Arango C Dragioti E Solmi M Cortese S Domschke K Murray RM Risk and protective factors for mental disorders beyond genetics: an evidence-based atlas. World Psychiatry 2021 20 417 36 10.1002/wps.20894 34505386 PMC8429329

[6] WHO and Calouste Gulbenkian Foundation. Social determinants of mental health. Geneva: *World Health Organization**;* *2014**. **Available from**:* https://apps.who.int/iris/bitstream/handle/10665/112828/9789241506809_eng.pdf. Accessed: May 6, 2024.

[7] Jones L Bellis MA Wood S Hughes K McCoy E Eckley L Prevalence and risk of violence against children with disabilities: a systematic review and meta-analysis of observational studies. Lancet 2012 380 899 907 10.1016/S0140-6736(12)60692-8 22795511

[8] Institute for Health Metrics and Evaluation. GBD Results Tool; 2019. In: Global Health Data Exchange [website]. Seattle: Institute for Health Metrics and Evaluation. Available from: https://ghdx.healthdata.org/gbd-results-tool?params=gbd-api-2019-permalink/d1089abd9ea6072c2e5203256b0c7960. Accessed: May 6, 2024.

[9] Cosma A, Abdrakhmanova S, Taut D, Schrijvers K, Catunda C, Schnohr C. A focus on adolescent mental health and wellbeing in Europe, central Asia and Canada. Health Behaviour in School-aged Children international report from the 2021/2022 survey. Volume 1. Copenhagen: WHO Regional Office for Europe; 2023. Licence: CC BY-NC-SA 3.0 IGO.

[10] Cosma A, Molcho M, Pickett W. A focus on adolescent peer violence and bullying in Europe, central Asia and Canada. Health Behaviour in School-aged Children international report from the 2021/2022 survey. Volume 2. Copenhagen: WHO Regional Office for Europe; 2024. Licence: CC BY-NC-SA 3.0 IGO.

[11] Burazeri G Goda A Tavanxhi N Sulo G Stefa J Kark JD The health effects of emigration on those who remain at home. Int J Epidemiol 2007 36 1265 72 10.1093/ije/dym162 17768161

[12] Burazeri G Goda A Sulo G Stefa J Kark JD Financial loss in pyramid savings schemes, downward social mobility and acute coronary syndrome in transitional Albania. J Epidemiol Community Health 2008 62 620 6 10.1136/jech.2007.066001 18559445

[13] Qirjako G Dika Q Mone I Draçini X Kuneshka L Roshi E Correlates of lifetime physical abuse among schoolchildren aged 15 years in post-communist Albania. Front Public Health 2021 9 607493 10.3389/fpubh.2021.607493 34395349 PMC8355483

[14] Inchley J, Currie D, Piper A, Jåstad A, Cosma A, Nic Gabhainn S, et al, editors. Health Behaviour in School-aged Children (HBSC) Study Protocol: background, methodology, mandatory questions and optional packages for the 2021/22 survey. MRC/CSO Social and Public Health Sciences Unit, The University of Glasgow; 2021/2022.

[15] Firth J Solmi M Wootton RE Vancampfort D Schuch FB Hoare E A meta-review of “lifestyle psychiatry”: the role of exercise, smoking, diet and sleep in the prevention and treatment of mental disorders. World Psychiatry 2020 19 360 80 10.1002/wps.20773 32931092 PMC7491615

[16] Firth J Siddiqi N Koyanagi A Siskind D Rosenbaum S Galletly C The Lancet Psychiatry Commission: a blueprint for protecting physical health in people with mental illness. Lancet Psychiatry 2019 6 675 712 10.1016/S2215-0366(19)30132-4 31324560

[17] Stubbs B Koyanagi A Hallgren M Firth J Richards J Schuch F Physical activity and anxiety: a perspective from the World Health Survey. J Affect Disord 2017 208 545 52 10.1016/j.jad.2016.10.028 27802893

[18] Stubbs B Vancampfort D Firth J Solmi M Siddiqi N Smith L Association between depression and smoking: a global perspective from 48 low-and middle-income countries. J Psychiatr Res 2018 103 142 9 10.1016/j.jpsychires.2018.05.018 29852421

[19] Vancampfort D Van Damme T Stubbs B Smith L Firth J Hallgren M Sedentary behavior and anxiety-induced sleep disturbance among 181,093 adolescents from 67 countries: a global perspective. Sleep Med 2019 58 19 26 10.1016/j.sleep.2019.01.048 31048258

[20] Wickrama KA Conger RD Lorenz FO Jung T Family antecedents and consequences of trajectories of depressive symptoms from adolescence to young adulthood: a life course investigation. J Health Soc Behav 2008 49 468 83 10.1177/002214650804900407 19181050 PMC2741725

[21] Fullana MA Tortella-Feliu M de la Cruz LF Chamorro J Pérez-Vigil A Ioannidis JP Risk and protective factors for anxiety and obsessive-compulsive disorders: an umbrella review of systematic reviews and meta-analyses. Psychol Med 2020 50 1300 15 10.1017/S0033291719001247 31172897

[22] World Health Organization. WHO European framework for action on mental health 2021–2025. Copenhagen: WHO Regional Office for Europe; 2022. Available from: https://apps.who.int/iris/handle/10665/352549. Accessed: May 6, 2024.

